# Radiation–Induced Signaling Results in Mitochondrial Impairment in Mouse Heart at 4 Weeks after Exposure to X-Rays

**DOI:** 10.1371/journal.pone.0027811

**Published:** 2011-12-08

**Authors:** Zarko Barjaktarovic, Dominik Schmaltz, Alena Shyla, Omid Azimzadeh, Sabine Schulz, Julia Haagen, Wolfgang Dörr, Hakan Sarioglu, Alexander Schäfer, Michael J. Atkinson, Hans Zischka, Soile Tapio

**Affiliations:** 1 Institute of Radiation Biology, Helmholtz Zentrum München, German Research Center for Environmental Health, Neuherberg, Germany; 2 Institute of Toxicology, Helmholtz Zentrum München, German Research Center for Environmental Health, Neuherberg, Germany; 3 Department of Radiotherapy and Radiooncology, Medical Faculty Carl Gustav Carus, University of Technology, Dresden, Germany; 4 Helmholtz Zentrum München, German Research Center for Environmental Health, Department of Protein Science, Proteomics Core Facility, Neuherberg, Germany; 5 Department of Radiation Oncology, Klinikum Rechts der Isar, Technische Universität München, Munich, Germany; University Health Network, Canada

## Abstract

**Backround:**

Radiation therapy treatment of breast cancer, Hodgkin's disease or childhood cancers expose the heart to high local radiation doses, causing an increased risk of cardiovascular disease in the survivors decades after the treatment. The mechanisms that underlie the radiation damage remain poorly understood so far. Previous data show that impairment of mitochondrial oxidative metabolism is directly linked to the development of cardiovascular disease.

**Methodology/Principal findings:**

In this study, the radiation-induced *in vivo* effects on cardiac mitochondrial proteome and function were investigated. C57BL/6N mice were exposed to local irradiation of the heart with doses of 0.2 Gy or 2 Gy (X-ray, 200 kV) at the age of eight weeks, the control mice were sham-irradiated. After four weeks the cardiac mitochondria were isolated and tested for proteomic and functional alterations. Two complementary proteomics approaches using both peptide and protein quantification strategies showed radiation-induced deregulation of 25 proteins in total. Three main biological categories were affected: the oxidative phophorylation, the pyruvate metabolism, and the cytoskeletal structure. The mitochondria exposed to high-dose irradiation showed functional impairment reflected as partial deactivation of Complex I (32%) and Complex III (11%), decreased succinate-driven respiratory capacity (13%), increased level of reactive oxygen species and enhanced oxidation of mitochondrial proteins. The changes in the pyruvate metabolism and structural proteins were seen with both low and high radiation doses.

**Conclusion/Significance:**

This is the first study showing the biological alterations in the murine heart mitochondria several weeks after the exposure to low- and high-dose of ionizing radiation. Our results show that doses, equivalent to a single dose in radiotherapy, cause long-lasting changes in mitochondrial oxidative metabolism and mitochondria-associated cytoskeleton. This prompts us to propose that these first pathological changes lead to an increased risk of cardiovascular disease after radiation exposure.

## Introduction

Adverse effects of ionizing radiation on the cardiovascular system have the potential for a large impact on public health. High doses of radiation applied to the heart during radiotherapy used in breast cancer [1]–[4], Hodgkin's disease [5] or childhood cancers [6] increase cardiovascular incidence and mortality. Epidemiological studies indicate that much lower irradiation doses [≤1 gray (Gy)] typical of occupational [7]–[12], medical [6], [13] or environmental exposures [14], [15] also increase the risk of cardiovascular disease (CVD) several decades after the exposure. However, this remains controversial as some studies find no association between low-dose ionizing radiation and an increased risk for CVD [16]–[22].

The molecular mechanisms underlying the development of radiation-induced heart disease are not well understood so far. It has been suggested that persistent changes in oxidative metabolism may mediate the responses to ionizing radiation, ultimately leading to inflammation and cardiovascular disease [23], [24]. Indeed, the data from survivors of the atomic bombings show enhanced persistent inflammation [25] and a radiation dose-dependent increase of vasculatory reactive oxygen species (ROS), even after adjustment for gender, age, smoking status and body mass [26]. The presence of long-lived clastogenic factors in the blood of individuals exposed to ionizing radiation has been shown in several studies [27]–[29]. Clastogenic factors are associated with oxidative stress and have the capacity to cause chromosomal breakage if transferred to cell cultures originating from non-irradiated individuals [27]–[29].

Mitochondria play a central role in oxidative metabolism, where the final products of glycolysis and fatty acid metabolism, pyruvate and acetyl CoA, are used in the Krebs cycle and by oxidative phosphorylation to produce energy. As heart tissue has a high energy demand, it is not surprising that mitochondria contribute about 40% of the total cellular volume of cardiomyocytes [30]. Approximately 90% of energy is supplied by these organelles [31]. In numerous biochemical and functional studies of cardiomyocytes, impairment of oxidative metabolism has been directly linked to the development of cardiovascular disease [24], [32]–[34]. Loss of control over the reduction and oxidation processes within the mitochondria may lead to disruption of metabolic homeostasis and an increased production of ROS such as peroxide, superoxide and hydroxyl radicals. Such an excess of ROS is capable of causing damage to many cellular components including lipids, proteins, and DNA [35], [36]. Oxidative stress is also known to contribute to vascular disease and endothelial cell dysfunction potentially leading to further cardiovascular damage [37]. Conversely, lower ROS concentrations stimulate cellular signaling and gene expression modulating vascular function [38] and playing an important role in cardioprotection [39], [40].

Exposure of eukaryotic cells to radiation leads to the production of ROS within minutes. Leach, et al. showed that, in the dose range between 1 and 10 Gy, the amount of ROS produced per cell was constant whereas the percentage of ROS producing cells increased with the dose [41]. This induced increase in ROS production was dependent on dysfunctional mitochondrial electron transport and was observed in several cell types.

We have shown previously that a total body irradiation (3 Gy gamma-ray) caused immediate (5 h, 24 h) increase in the level of protein oxidation and lipid peroxidation in the cardiac tissue of C57BL/6 mice [42]. Mitochondrial proteins represented the protein class most sensitive to ionizing radiation. Whether an immediate burst of ROS may lead to persistent alterations in cells and tissues after days and weeks is unknown.

The goal of this study was to determine whether ionizing radiation causes non-transient impairment of cardiac mitochondria that could finally lead to cardiovascular disease. For this purpose C57BL/6N mice were locally irradiated to the heart using an acute dose of 0.2 Gy or 2 Gy X-ray; the control mice were sham-irradiated. These doses were chosen while the higher dose equivalent (2 Gy) is frequently used as a single dose in the radiation therapy. Epidemiological data clearly show a relation between this dose and increased risk for CVD [1]–[4]. However, it is uncertain whether doses below 0.5 Gy have an impact on CVD risk [16], [17]. The radiation-induced effects on cardiac mitochondria were investigated four weeks after irradiation to observe the first persistent pathological changes.

We used two complementary quantitative proteomic approaches, ICPL and 2D-DIGE; ICPL is peptide-based and 2D-DIGE protein-based quantification of proteome changes. Using LC-ESI/MS/MS identification of the deregulated proteins and bioinformatics analysis we were able to elucidate a radiation-induced mitochondrial impairment *in vivo*. Based on the proteomics data we performed functional studies of isolated mitochondria from mouse hearts on complex activity, respiration, ROS generation and global proteome oxidative status. We show that intracellular changes caused by initial radiation-induced oxidative stress persist over a long period of time, with potential accumulative effects.

## Materials and Methods

### Local cardiac irradiation of mice

All animals were treated in compliance with the German animal welfare law and the experiments were approved by the institutional committee on animal experimentation and the government of Upper Bavaria (Certificate of Landesdirektion Dresden 24(D)-9168.11-1-2008-10).

Breeding stock C57BL/6N mice were originally purchased from Charles River Laboratories, Germany GmbH. The animals were bred and housed under specified pathogen-free conditions with controlled conditions of temperature (21–24°C) and humidity (30–50%). An automated light program regulated a 12/12-h light/dark rhythm, with lights on from 06:00 a.m. to 06:00 p.m.

Mice were housed in size 3 Macrolon® cages, maximum of 10 mice per cage, on sawdust bedding (Lignocel 3/4 S, ssniff Spezialdiäten GmbH, Germany). The animals had free access to standard mouse diet (ssniff® R/M-H, ssniff Spezialdiäten GmbH, Germany) and filtered city tap water.

For the local irradiation of the heart an YXLON MG325 X-ray apparatus (Yxlon International X-ray GmbH, Germany) was operated at 200 kV, with a tube current of 20 mA and a beam filter of 0.6 cm Cu, resulting in a dose rate of ca. 0.8 Gy/min at the focus-to-object-distance of 44.6 cm. Male animals at an age of 8±1 week were used for all experiments. The mice were immobilized (without anesthesia) in specially designed jigs. Six animals were irradiated simultaneously, with the 6 jigs arranged circularly on a perspex plate. The dose homogeneity between the individual heart irradiation fields was <3%. The treatment fields of 9.7×14 mm^2^ were defined by windows in a collimator plate consisting of 6 mm lead and 2 mm aluminium.

Before irradiation, the correct position of the hearts was verified by digital radiographs, resulting in an additional total body dose of 4 mGy. Subsequently, single local heart doses of 0.2 Gy or 2 Gy were applied. The control sham-irradiated group was treated similarly as the exposed groups except that the irradiation source was not turned on.

The total number of C57BL/6N mice used in this study was 51.

### Isolation of cardiac mitochondria

After sacrificing mice by cervical dislocation, the hearts were rapidly removed, rinsed and finally minced on ice in isolation buffer [0.3 M sucrose (Fluka), 5 mM N-[Tris(hydroxymethyl)methyl]-2-aminoethanesulfonic acid, 0.2 mM EGTA, pH 7.2]. The minced tissue was gently homogenized in a roughened glass homogenizer (FORTUNA, Poulten & Graf GmbH) with 4–5 strokes. Homogenates were centrifuged at 1400 *g*, 4°C for 10 min and resulting supernatants were re-centrifuged at 9000 g, 4°C for 10 min to receive the mitochondrial pellet. It was washed once with isolation buffer and centrifuged again at 9000 *g*, 4°C for 10 min. Protein concentration was determined by Bradford Reagent (Sigma) with bovine serum albumin as standard. For all measurements isolated mitochondria were kept on ice and used within 3–4 hours.

To obtain a high-purity mitochondrial fraction for proteomic analysis, differential centrifugation technique and discontinuous Percoll density gradient were used [43], [44]. Mitochondrial marker VDAC was enriched in the mitochondrial fraction as compared to the whole heart homogenate, while a protein marker of endoplasmic reticulum BiP was barely detectable (Information S1). In addition, the purity of mitochondrial preparations was determined by electron microscopy (Information S1). Intactness and functionality of isolated mitochondria were further analyzed by measuring the respiration capacity of each mitochondrial sample. For proteomic analysis mitochondrial fractions were precipitated by 2D clean up kit (Bio-Rad) to remove lipids, nucleotides, and salts.

### Proteomic analysis

#### ICPL Labeling and 1DE

The three biological replicates were labeled with ICPL reagent (SERVA) as described previously [45], [46]. Triplicate aliquots of 100 µg of mitochondrial proteins obtained from heart tissue of sham- or irradiated mice were precipitated with the 2D clean-up kit (GE Healthcare) following the manufacturer's instructions. Protein quantification was done from the pellet in triplicate using both Bradford method and 2D Quant Kit (GE Healthcare) following the manufacturer's instructions. Both sham-irradiated and irradiated protein samples were individually alkylated and acylated as follows. To 40 µL of each solution, 1 µl of 0.2 M TCEP (Tris-2-carboxyethyl phosphine) was added, and the disulphide bonds were reduced for 30 min at 60°C. After cooling down, all free thiol groups of the cysteines were alkylated with 1 µl of 0.4 M iodoacetamide for 30 min at room temperature in the dark. Excess iodoacetamide was quenched by adding 1 µl of 0.5 M *N*-acetylcysteine. For nicotinoylation, a ten-fold molar excess of all free amino groups of ^12^C- and ^13^C-nicotinoyloxysuccinimide (Nic-NHS) (ISOTEC, Miamisburg, USA), was added to the sham and exposed samples, respectively, and the reaction was allowed to proceed for 2 h at pH 8.3 at room temperature. 4 µl of 1.5 M hydroxylamine was added to each sample to destroy the remaining Nic-NHS reagents. Esters, which form during the labeling procedure, were hydrolyzed by raising the pH to 11–12 for 20 min. Equal amounts of light and heavy labeled samples were combined and separated by 12% SDS gel electrophoresis [47] before staining with colloidal Coomassie [48]. The gel lanes were cut in 2 slices and subjected to in-gel digestion. To validate the accuracy of the ICPL labeling, 3 µg of protein test mixtures, each containing different amounts of 3 proteins (BSA with a light to heavy ratio 1:1; ovalbumin 4:1; carbonic anhydrase 1:2 were mixed with the samples.*2D-DIGE analysis*

Mitochondria from sham-irradiated and irradiated murine hearts were isolated from five independent biological samples with two technical replicates in every group and labeled with CyDye™ DIGE Fluor minimal dyes (400 pmol/50 µg) (GE Healthcare), according to the manufacturer's recommendations. Isoelectric focusing (IEF) was performed using immobilized pH gradients (24 cm; pH 3–10 nonlinear range; GE Healthcare). 50 µg of protein in rehydration buffer (8 M urea, 2% CHAPS, 30 mM DTT, 0.5% ampholines, pH 4–7) was applied using cup loading (GE Healthcare). IEF was carried out at 20°C, using voltages and running times as follows: 12 h passive rehydration, rapid 300 V for 3 h, gradients from 300 to 1000 V for 3 h, 1000 to 3500 V for 2 h, 3500 to 10000 V for 3 h and finally rapid 10 000 V up to a total of 75 kVh). The maximum current was 50 µA per gel strip. Gel strips were incubated in equilibration solution (50 mM Tris/HCl, pH 8.8, 6 M urea, 2% (w/v) SDS, 1% (w/v) DTT) for 15 min, followed by 15 min incubation with this solution with DTT substituted by 2.5% (w/v) iodoacetamide). Equilibrated gel strips were placed on top of a 12% acrylamide gel and overlaid with 0.5% agarose solution. SDS-PAGE was carried out using the Ettan DALTtwelve system (GE Healthcare), and performed at 1 W/gel for 1 h, followed by 15 W/gel for 5 h.

#### Image analysis

Gels were scanned at a resolution of 100 µm on a Typhoon 9400 (GE Healthcare). Cy2-, Cy3- and Cy5-Dye images of each gel were acquired at excitation/emission values of 488/520, 523/580 and 633/670nm, respectively. After image acquisition, the gels were fixed overnight in 30% ethanol and 10% acetic acid, and stored at 4°C. For mass spectrometry-based identification the gels were post-stained with silver according to Chevallet, et al. [49]. Scanned images were cropped using the ImageQuant software version 5.2 (GE Healthcare). The DeCyder version 6.5 software (GE Healthcare) was used for image analysis. The Differential In-gel Analysis (DIA) module was used for automatic spot detection. Abundance measurements for each individual gel were obtained by comparing the normalized volume ratio of each spot from a Cy3- or Cy5-labeled sample to the corresponding Cy2-signal from the pooled-sample internal standard. The DIA datasets from each individual gel were collectively analyzed using the Biological Variation Analysis (BVA) module, which allows inter-gel matching and the calculation of the average abundance for each protein spot among the five gels of each group. Statistical significance was assessed for each change in abundance using Student's t-test. Five biological with two technical replicates of each were used for statistical analysis. We considered spots as differentially regulated if statistical significance at the 95% confidence level was achieved and if the standardized average spot volume ratio exceeded 1.30-fold. Calculation of experimental MW and pI for each differential protein spot was done with the help of the given pH range of the IPG-strips and with externally applied molecular weight marker proteins. False Discovery Rate (FDR) correction was applied in the statistics. Proteins with statistically significant differential expression (Student's t-test p<0.05 and fold difference >1.30 or <0.770) were manually picked from 2D-DIGE gels post-stained with silver.

##### *In-gel Digestion*

1D stained gel slices or 2D spots were excised from polyacrylamide gels and subjected to in-gel digestion before MS analysis. The silver stained 2D gel spots were transferred to protein low bind tubes, and destained with 15 mM K_3_Fe(CN)_6_ and 50 mM Na_2_S_2_O_3_. The gel pieces were washed once with 500 µl water and twice with 200 mM NH_4_HCO_3_ for 15 min_._ The liquid was removed and the proteins were reduced and alkylated, except for 2D gel spots. The gel pieces were washed with water and shrunk with 25 µl acetonitrile (ACN) for 5 min. Subsequently, the gel pieces were dried and the samples were rehydrated with 10 µl of trypsin (Promega, 10 µng/µl in 50 mM NH_4_HCO_3_). After 10 min the gel pieces were covered with 10 to 30 µl (until the spots were completely covered with liquid) of 50 mM NH_4_HCO_3_ and digested O/N at 37°C. The resulting peptide mixture was extracted twice with 50 µl of 50% ACN, 2.5% TFA by sonication for 10 min. The supernatants were collected in a fresh protein low bind tube, frozen in liquid nitrogen and reduced to a volume of 10 to 20 µl in a speedvac.

#### LC/MS/MS Analysis

The digested peptides were separated by reversed phase chromatography (PepMap, 15 cm×75 µm ID, 3 µm/100Å pore size, LC Packings) operated on a nano-HPLC (Ultimate 3000, Dionex) with a nonlinear 170 min gradient using 2% acetonitrile in 0.1% formic acid in water (A) and 0.1% formic acid in 98% acetonitrile (B) as eluted with a flow rate of 250 nl/min. The gradient settings were subsequently: 0–140 min: 2–30% B, 140–150 min: 31–99% B, 151–160 min: Stay at 99% B and equilibrate for 10 min at starting conditions. The nano-LC was connected to a linear quadrupole ion trap-Orbitrap (LTQ Orbitrap XL) mass spectrometer (Thermo Fisher, Bremen, Germany) equipped with a nano-ESI source. The mass spectrometer was operated in the data-dependent mode to automatically switch between Orbitrap-MS and LTQ-MS/MS acquisition. Survey full scan MS spectra (from m/z 300 to 1500) were acquired in the Orbitrap with resolution R = 60,000 at m/z 400 (after accumulation to a target of 1,000,000 charges in the LTQ). The method used allowed sequential isolation of up to ten most intense ions depending on signal intensity, for fragmentation on the linear ion trap using collision-induced dissociation at a target value of 100,000 ions with a normalized collision energy of 35% and an activation time of 30 ms. Minimum signal intensity required was 200, isolation width 2 amu and default charge state 2. Precursor masses were selected in a data-dependent manner. High resolution MS scans in the Orbitrap and MS/MS scans in the linear ion trap were performed in parallel. Target peptides already selected for MS/MS were dynamically excluded for 30 seconds. General mass spectrometry conditions were: electrospray voltage, 1.25–1.4 kV; no sheath and auxiliary gas flow. An activation Q-value of 0.25 and activation time of 30 ms were also applied for MS/MS. The acquired MS/MS spectra were searched against the UniRef100 database (date: 20100729, number of residues: 3761183040, number of sequences: 10711464); the number of sequences for taxonomy *Mus musculus* (house mouse) is 84975. We used a version of MASCOT (Matrix Science, version 2.3.02) with the following parameters: MS/MS spectra were searched with a precursor mass tolerance of 10 ppm and a fragment tolerance of 0.8 Da. MASCOT scores are probability-based MOWSE score: –10xLog(*P*), where *P* is the probability that the observed match is a random event. Scores >34 indicate identity or extensive homology; *p* <0.05. MASCOT peptide scores are shown in the Information S2. One missed cleavage was allowed. Carbamidomethylation was set as fixed modification. Oxidized methionine and the heavy and light ICPL labels of lysines as well as heavy and light ICPL labels of the protein N-terminus were set as variable modifications.

Scaffold (version Scaffold_3_00_07, Proteome Software Inc., Portland, OR) was used to validate MS/MS-based peptide and protein identifications. Peptide identifications were accepted if they could be established at greater than 80.0% probability as specified by the Peptide Prophet algorithm [50]. Protein identifications were accepted if they showed greater than 95.0% probability and contained at least 2 identified peptides. Protein probabilities were assigned by the Protein Prophet algorithm [51]. Proteins that contained similar peptides and could not be differentiated based on MS/MS analysis alone were grouped to satisfy the principles of parsimony. All proteins showing the following criteria calculated by Proteome Discoverer (Thermo Scientific) and Perseus software tool [52]: significance p <0.05; fold-change>1.3 or <0.770; variability <15% and were quantified by two unique peptides were considered as deregulated.

Detailed experimental settings are described in Information S2 (Sheet Experimental settings).

##### MALDI TOF/TOF protein identifications

0.5 µl of sample was spotted onto a stainless steel MALDI target plate by the dried droplet method. The matrix used was 3.75 mg/ml α-cyano-4-hydroxycinnamic acid in 60% ACN, 0.1% TFA.

Mass spectra were acquired using a 4700 Proteomics Analyzer (MALDI-TOF-TOF) (Applied Biosystems). Measurements were performed with a 355 nm Nb:YAG laser in positive reflector mode with a 20 kV acceleration voltage. The mass range (m/z 900–4000) was externally calibrated using the peptide calibration standard III (Applied Biosystems). For each MS and MS/MS spectrum 3000 laser shots were accumulated. Spectra acquisition and processing was done in automatic mode with 4000 Series Explorer software (version 3.6, Applied Biosystems).

The GPS Explorer ™ Software (version 3.6, Applied Biosystems) was used for spectra analyses. The database search was performed with MASCOT (Version: 2.2.06) using the mouse UniRef100 20090718 (selected for *Mus musculus,* 78401 entries) and Swiss-Prot database (Swiss-Prot version from 20090212). One missed trypsin cleavage was selected. Carbamidomethylation was set as the fixed modification and oxidized methionine as the variable modification. Precursor tolerance was set to 75 ppm and MS/MS fragment tolerance to 0.3 Dalton. The shown MASCOT protein scores are a summary of scores for each MS/MS spectra and an additional score for the peptide mass fingerprint. The significance level (p-value <0.05) for a protein score is usually higher than a MASCOT score of 50–60 (for an analysis of this dataset against Swiss-Prot this corresponds to a MASCOT score >56).

#### Analysis of the signaling network of deregulated proteins

The analyses of protein-protein interaction and signaling networks were performed by the software tool INGENUITY Pathway Analysis (IPA) (INGENUITY System, http://www.INGENUITY.com). IPA is a knowledge database generated from peer-reviewed scientific publications that enables discovery of highly represented functions and pathways (*p* <0.001) from large, quantitative data sets [53], [54]. The analysis provides the information of relationships, biological mechanisms, functions, and pathways of relevance associated with the identified proteins. The protein accession numbers including the relative expression values (fold change) of each protein were uploaded for the core analysis.

The Fischer's exact test was used to calculate a *p*-value determining the probability that each biological function or disease assigned to that network is due to a random event. The score for each network is a numerical value to approximate the degree of relevance and size of a network to the molecules in the given dataset.

### Immunoblotting analysis

For the validation of protein expression changes by immunoblotting [55], 20 µg of mitochondrial extract was separated on 8% and 12% SDS polyacrylamide gels according to Laemmli [56]. Proteins were transferred to nitrocellulose membranes (GE Healthcare) using a semidry blotting system at 100 mA for 90 min. Membranes were saturated for one hour with 5% advance blocking reagent (GE Healthcare) in TBS (50 mM Tris.HCl, pH 7.6 and 150 mM NaCl) containing 0.1% Tween 20 (TBS/T). Blots were then incubated overnight at 4°C with antibodies against either cytochrome c1 (Abnova), pyruvate dehydrogenase E1α with α-tubulin as the loading control (all from Sigma-Aldrich). To compare the amounts of mitochondrial respiratory complexes in sham vs. irradiated mitochondria we used a premixed cocktail including one antibody against each complex: CI subunit NDUFB8, CII-30kDa, CIII-Core protein 2, CIV subunit I and CV alpha subunit (MitoSciences, USA). After washing three times in TBS/T, blots were incubated for one hour at room temperature with horseradish peroxidise-conjugated anti-mouse or anti-goat secondary antibody (Santa Cruz Biotechnology) in blocking buffer (TBS/T with 5% w/v advance blocking reagent). Immunodetection was performed with ECL advance Western blotting detection kit (GE Healthcare). The protein bands were quantified using ImageQuant 5.2 software (GE Healthcare) by integration of all pixel values in the band area after background correction, and normalized to the α-tubulin expression.

### Complex I, Complex III, pyruvate dehydrogenase and aconitase activities

Complex I and pyruvate dehydrogenase activity was quantified using the dipstick assay kit according to the manufacturer's recommendations (Mitoscience). The enzymes were immunocaptured in an active form on a dipstick using anti-Complex I and anti-pyruvate dehydrogenase monoclonal antibodies. Then dipstick was immersed in Complex I activity buffer, containing NADH as a substrate and nitrotetrazolium (NTB) as the electron acceptor. Immocaptured Complex I reduced NBT to form blue-purple precipitate at the Complex I antibody line. Pyruvate dehydrogenase activity was visualized as reduction of NBT in the presence of excess diaphorase. The signal intensity, corresponding to the enzyme activity, was analyzed using Image Quant 5.2 software (GE Healthcare).

Complex III (ubiquinol-cytochrome c reductase) activity was determined by measuring the reduction of cytochrome c at 550 nm as described by Kiebish, et al. [57]. Briefly, the Complex III assay was performed in buffer containing 25 mM potassium phosphate, pH 7.4, 1 mM EDTA, 1 mM KCN, 0.6 mM dodecyl maltoside, and 32 µM oxidized cytochome c, using 2 µg of crude mitochondria. The reaction was initiated with 35 µM decylubiquinol and the enzyme activity was quantified by following the reaction for 1 min both in the presence and absence of 2 µM antimycin (inhibitor). Decylubiquinol was freshly made by dissolving 10 mg of decylubiquinone in 2 ml acidified ethanol (pH 2) and using sodium dithionite as a reducing agent. Decylubiquinol was further purified by cyclohexane [58]. Four biological replicates were used for statistical analysis.

Aconitase activity was measured according to manufacturer recommendations (Cayman, USA). Reaction was initiated with isocitrate with and without inhibitor (oxalomalate) and monitored by measuring the formation of NADPH at 340 nm for 10 minutes.

### Analysis of free radical content

Mitochondria at a protein concentration of 7.5 µg/µl in isolation buffer were incubated with 2 mM 2′,7′-dichlorodihydrofluorescein diacetate (H_2_DCFDA) (Invitrogen) for 10 min on ice with subsequent centrifugation at 9000 g for 10 min at 4°C. After a washing step the pellet of labelled mitochondria was resuspended in swelling buffer (SwB) containing 0.2 M sucrose, 10 mM MOPS-Tris, 5 mM succinate, 1 mM Pi , 10 µM EGTA, and 2 µM rotenone and 10 µl aliquots (75 µg mitochondrial sample per well) were added to SwB in a black flat bottom 96 well plate. Directly before measuring the fluorescence 50 µl stimuli were added to give a final volume of 200 µl per well. Measurements of fluorescence were performed (BioTek® Instruments Inc.) with an excitation wavelength 485/20 nm and an emission wavelength of 528/20 nm at room temperature for 60 min. In the presence of 5 µM succinate, 50 µM mercury (II)acetate or 100 µM *tert*-butyl hydroperoxide were added to stimulate the production of ROS. All measurements were done in duplicates. To compare the production of free radicals produced by mitochondria isolated from irradiated and sham-irradiated hearts the area under the curve (AUC) was calculated using the software GraphPad Prism 4 (GraphPad Software, Inc.) for at least 5 biological replicates. Statistical comparisons were made using the nonparametric Mann-Whitney test. Results were considered significant at p <0.05.

### Saturation DIGE labeling of oxidized mitochondrial proteins

Saturation DIGE labeling was carried out according to manufacturer's protocol, except that reduction step tris(2-carboxyethyl)phosphine prior labeling was omitted in order to keep the cysteins in their oxidized state [59]. Since this reagent label specifically free thiol groups, this analysis will address exclusively the oxidation status of cysteine residues in the proteins. Briefly, protein pellets were first resuspended in 8 M urea, 4% CHAPS and 30mM Tris at pH 8.0. After pH adjustment, five µg of protein was labeled with 5 nM Cye5 (2 Gy) and Cye3 (control) dyes 1 h at 37°C. As additional control, the same samples were labeled using Cye 3 dye in order to avoid changes due to the different channels of scanning, and loaded into different wells on 1D gels. Prior labeling samples were overlayed with nitrogen. Throughout the procedure samples were kept with the minimum of light exposure. The reaction was quenched by adding the same volume of 8 M urea, 4% CHAPS and 2% DTT. After 1D SDS PAGE, the gels were scanned using Typhoon scanner and bands quantified after background subtraction and normalized to post-stained silver gels using Image Quant software.

### Mitochondrial respiration

Mitochondrial respiration was measured at 30°C using a Hansatech oxygen electrode (Hansatech Instruments) in 500 µl respiratory media containing 0.14 M mannitol, 0.05 M sucrose, 10 mM phosphate buffer (pH 7.4), 5 mM MgCl_2_, 2 mM Tris/HCl (pH 7.4), 0.25 mM EDTA (pH 7.8) as described previously [60]. Respiration was measured using a range of substrates that enter metabolic pathways at different locations. For palmitoyl carnitine /malate respiration measurements substrates were added to final concentrations of 40 µM and 5 mM, respectively [61]. Complex I was blocked by adding 12 µM rotenone and respiration was initiated by adding 10 mM succinate. Subsequently, 1 mM ADP and later 56 µM oligomycin were added. The quality of isolated mitochondrial preparations was assessed by calculation of respiratory control ratios RCR_S_ (rate of ADP-dependent respiration – rate of rotenone-dependent respiration / rate of succinate-dependent respiration – rate of rotenone-dependent respiration) to ensure that only highly coupled mitochondrial samples were used [61], [62]. Respiration was measured using 3 technical replicates from at least 5 biological samples. Respiratory rates after addition of the substrates were used for the comparison of mitochondria isolated from sham-irradiated and irradiated C57BL/6N mice.

### Electron microscopy

Freshly isolated cardiac mitochondria (100–200 µg) were immediately pelleted by centrifugation at 4°C (10 min at 9000 g), fixed in 2.5% glutaraldehyde in 0.1 M sodium cacodylate buffer (pH 7.4), postfixed with 1% osmium tetroxide, dehydrated with ethanol, and embedded in Epon. Ultrathin sections were negatively stained with uranyl acetate and lead citrate and then analyzed on a Zeiss EM 10 CR electron microscope.

## Results

### Mitochondrial proteomics revealed radiation-induced deregulation of 25 proteins

Effects of localized irradiation (2 Gy and 0.2 Gy) of the heart were analyzed using two proteomic approaches: ICPL and 2D-DIGE. Using the ICPL method, the number of identified, quantified and deregulated proteins was 635, 303 and 15, and 778, 421 and 5 with doses of 2 Gy and 0.2 Gy, respectively. Using the 2D-DIGE method, an average of 930 spots was detected of which seven proteins in total showed significant deregulation (0.2 Gy and 2.0 Gy). Taken together, the ICPL method was more sensitive as seen with the number of deregulated proteins whereas only 2D-DIGE was able to detect protein isoforms, fragments and modified proteins.

### ICPL

ICPL was performed in biological triplicates using duplex labeling, followed by 1D gel separation and subsequent LC-MS/MS analysis. As shown in the Information S1, the protein standards premixed with the samples showed expected abundance ratios (carbonic anhydrase II, 2.08; ovalbumin, 0.244; serum albumin 0.93; Information S1) with relative error well below 10%, demonstrating the accuracy of the analytical method. Irradiation of the heart with the 2 Gy dose induced changes in 15 mitochondrial proteins. Six proteins were significantly up-regulated (>1.3-fold) (p ≤0.050) [52], and nine were down-regulated (<0.770-fold) (Table 1).Lower dose (0.2 Gy) induced significant changes in five proteins (three up- and two down-regulated; Table 2). Deregulated proteins were quantified in Proteome Discoverer software using at least two unique peptides. Cytochrome c oxidase polypeptide 7A1 was quantified by one unique peptide and was manually validated.

**Table 1:** Differentially regulated proteins after 2.0 Gy irradiation using ICPL approach.

AccessionUniProt	IPAGene name	MW [kDa]	calc. pI	Protein name	ΣCoverage/%	Σ# Peptides	ΣQuantified peptides in replicates	Fold change(Heavy/Light)	Fold changeVariability [%]
P63268	ACTG2	41.8	5.48	Actin, gamma-enteric smooth muscle	31.65	6	2;2;1	**2.476**	6.8
Q6P8P3	MYH6	223.4	5.73	Myosin-6	45.25	59	38;46;3	**1.626**	5.9
Q6P8J7	CKMT2	47.4	8.40	Creatine kinase, sarcomeric mitochondrial	63.96	18	47;52;35	**1.585**	2.7
P48962	SLC25A4	32.9	9.72	ADP/ATP translocase 1	52.68	9	22;24;12	**1.472**	5.6
Q9CR62	SLC25A11	34.1	9.94	Mitochondrial 2-oxoglutarate/malate carrier protein	35.35	10	7;10;4	**1.441**	0.8
Q9Z2Z6	SLC25A20	33.0	9.11	Mitochondrial carnitine/acylcarnitine carrier protein	11.63	2	1;1;1	**1.362**	5.0
Q91WS0	CISD1	12.1	9.06	CDGSH iron sulfur domain-containing protein 1	60.19	2	2;2;1	**0.499**	4.0
Q9DCC8	TOMM20	16.3	8.60	Mitochondrial import receptor subunit TOM20 homolog	6.90	2	2;3;2	**0.558**	5.5
Q8R1I1	UQCR10	13.01	8.86	Cytochrome b-c1 complex subunit 9	57.81	2	1;1;1	**0.600**	2.2
P47738	ALDH2	56.5	7.62	Aldehyde dehydrogenase, mitochondrial	26.40	7	3;5;1	**0.611**	8.0
O35143	ATPIF1	12.2	9.64	ATPase inhibitor, mitochondrial	34.91	3	2;5;2	**0.632**	2.2
Q9WUM5	SUCLG1	35.0	9.39	Succinyl-CoA ligase [GDP-forming] subunit alpha, mitochondrial	10.51	2	4;3;3	**0.661**	2.0
Q8BK30	NDUFV3	50.5	8.97	NADH dehydrogenase [ubiquinone] flavoprotein 3, mitochondrial	20.09	4	2;2;-	**0.668**	7.7
Q62425	NDUFA4	9.3	9.52	NADH dehydrogenase [ubiquinone] 1 alpha subcomplex subunit 4	57.32	2	-;2;2	**0.722**	9.3
P56392	COX7A1	9.0	9.79	Cytochrome c oxidase polypeptide 7A1, mitochondrial	36.25	1	2;3;3	**0.755**	1.6

Mitochondrial proteins from 2 Gy-irradiated and sham-irradiated hearts were labeled with the heavy and light isotopes, respectively. The proteins demonstrated in the table were identified by two or more unique peptides. Theoretical isoelectric points (pI) and molecular weights (Mw) are derived from the amino acid sequences in Swiss-Prot. All proteins showing significant (p <0.05; Perseus Statistics program) upregulation (>1.3-fold) or downregulation (<0.770-fold) were considered as deregulated. Replicate protein ratios were averaged to take account for biological variability. Spread of the ratios for one protein over biological replicates is given as %CV (Fold change Variability).

**Table 2:** Differentially regulated proteins after 0.2 Gy irradiation using ICPL approach.

AccessionUniProt	MW [kDa]	calc. pI	Protein Name	ΣCoverage/%	Σ# Peptides	ΣQuantified peptides in replicates	Fold change(Heavy/Light)	Fold change Variability [%]
P20152	53.7	5.12	Vimentin	41.42	14	4;5;5	**1.365**	1.0
Q62425	9.3	9.52	NADH dehydrogenase [ubiquinone] 1 alpha subcomplex subunit 4	57.32	2	2;3;1	**1.360**	6.6
P31001	53.5	5.27	Desmin	55.65	18	7;8;7	**1.348**	4.0
Q8BMF3	67.1	7.83	NADP-dependent malic enzyme, mitochondrial	17.22	6	1;1;1	**0.688**	12.6
Q3TFD0	55.7	8.47	Serine hydroxymethyltransferase 2, mitochondrial	7.34	3	-;1;1	**0.714**	3.7

Mitochondrial proteins from 0.2 Gy-irradiated and sham-irradiated hearts were labeled with the heavy and light isotopes, respectively. The proteins demonstrated in the table were identified by two or more unique peptides. Theoretical isoelectric points (pI) and molecular weights (Mw) are derived from the amino acid sequences in Swiss-Prot. All proteins showing significant (p <0.05; Perseus Statistics program) upregulation (>1.3-fold) or downregulation (<0.770-fold) were considered as deregulated. Replicate protein ratios were averaged to take account for biological variability. Spread of the ratios for one protein over biological replicates is given as %CV (Fold change Variability).

### 2D-DIGE

**Figure 1:**
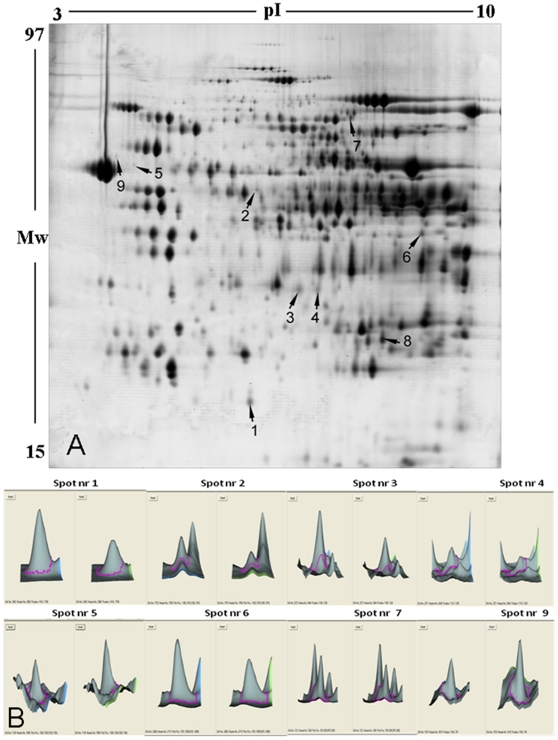
Two-dimensional separation of proteins from mouse heart mitochondria using DIGE approach. **A.** Indicated spots show significant increase or decrease in expression (t-test, p≤0.05) after image analysis in DeCyder software (GE Healthcare). Five independent biological and two technical replicates of each were used for the statistical analysis. **B.** The three-dimensional structure of spots (left sham, right 2 Gy) with numbers 1, 2, 3, 4, 5, 6, 7, 9 are shown.

The image analysis of 2 Gy- vs. sham-irradiated mitochondria revealed five protein spots significantly increased/decreased in amount by 1.5-/0.667-fold (t-test; p<0.001; n = 5). By lowering the threshold to 1.3-/0.770-fold, four additional spots appeared to be significantly deregulated after irradiation (p<0.001; Figure 1, Information S1). The lower dose (0.2 Gy) induced a significant decrease in the amount of protein spot (number 2) with a similar down-regulation as after the high dose exposure (0.581/0.2 Gy vs. 0.613/2 Gy), indicating a dose-independent response. In contrast, spots 3 and 4 were down-regulated with both doses but to a lesser degree with the low dose than with the high dose, indicating a dose-dependent response.

Four spots representing abundant proteins (spots 3, 4, 7, 8; Figure 1) were successfully identified using the MALDI TOF/TOF technique. Low abundance spots were identified by LC-MS/MS using the Orbitrap XL. Peptides corresponding to deregulated proteins detected by 2D-DIGE approach and identified by LC-MS/MS (Orbitrap XL) or MALDI-TOF/TOF are shown in Information S2. Table 3 shows the protein identifications according to their highest homologies with fragment pattern reported for known proteins for *Mus musculus*. Categorized by the biological function the identified proteins belong to the electron transport chain (ETC) (cytochrome c1 as two distinct spots on the gel), glycolysis [pyruvate dehydrogenase subunit E1 α (PDH E1α), lipid metabolism (succinyl-CoA:3-ketoacid-coenzyme A transferase 1; long-chain-fatty-acid–CoA ligase**;** succinyl-CoA:3-ketoacid-coenzyme A transferase 1) or cellular structure (vimentin, desmin). Proteins found to be significantly down-regulated represented metabolic pathways (cytochrome c1, PDH subunit E1α, succinyl-CoA:3-ketoacid-coenzyme A transferase 1, long-chain-fatty-acid–CoA ligase). The structural proteins vimentin and desmin that support mitochondrial morphology and organization, and a protein of unknown function (ES1 homolog) were significantly up-regulated after the high-dose radiation exposure.

**Table 3:** Differentially regulated proteins after 2.0 Gy and 0.2 Gy irradiation using 2D-DIGE.

Spot number	AccessionUniProt	IPAGene name	MW[kDa]	calc. pI	Protein name	ΣCoverage/%	Σ# Peptides	Fold change2.0 Gy/sham	Fold change0.2 Gy/sham
9	P20152	VIM	53.7	5.06	Vimentin	48	23	**1.600**	1.170
5	P31001	DES	53.5	5.21	Desmin	34	16	**1.400**	1.020
8	Q9D172	C21orf33	28.1	9.00	ES1 protein homolog, mitochondrial	33	7	**1.330**	0.952
1	Q3U9P7	OXCT1	56.0	8.73	Succinyl-CoA:3-ketoacid-CoA transferase 1	11	5	**0.513**	N.F.
2	P35486	PDHA1	42.2	8.49	Pyruvate dehydrogenase E1 component subunit alpha	34	13	**0.613**	**0.581**
4	Q9D0M3	CYC1	35.5	9.24	Cytochrome c1, heme protein, mitochondrial	40	11	**0.625**	0.840
3	Q9D0M3	CYC1	35.5	9.24	Cytochrome c1, heme protein, mitochondrial	43	16	**0.662**	0.826
6	Q9CRF4	OXCT1	29.4	8.97	Succinyl-CoA:3-ketoacid-coenzyme A transferase 1	47	8	**0.752**	1.140
7	P41216	ACSL1	78.1	6.81	Long-chain-fatty-acid–CoA ligase	31	12	**0.770**	N.F.

The fold changes indicated in bold are considered significantly deregulated (>1.3 or <0.77). Spots with numbers 3, 4, 6, 7 were identified by MALDI-TOF/TOF. Spots with numbers 1, 2, 5, 8 and 9 were identified by LC-MS/MS (Orbitrap XL). All proteins showing significant (p<0.05) upregulation (>1.3-fold) or downregulation (<0.770-fold) were considered as deregulated. MW, molecular mass of predicted proteins; pI, isoelectric point of predicted proteins; sequence coverage (%), percentage of predicted protein sequence covered by matched peptides; no. matched, number of peptides matched. N.F.; not found.

Taking into account that some proteins were identified as deregulated with both methods or from several 2D-DIGE spots, the total number of differentially regulated proteins after high or low radiation exposure was 25.

### In silico pathway analysis of deregulated proteins ascertained the mitochondrial Complex III as a radiation target

**Figure 2:**
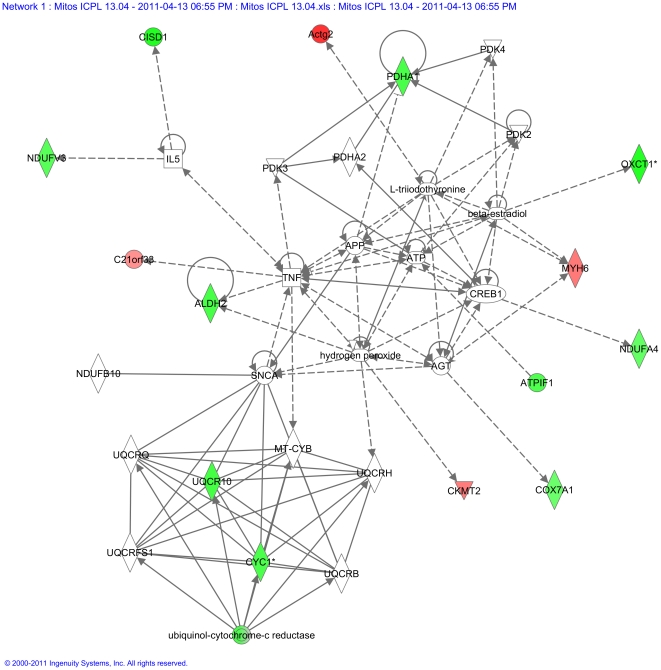
Graphical representation of the top protein interaction network of differentially regulated proteins. All deregulated proteins (0.2 Gy and 2 Gy ICPL and 2D-DIGE; Tables 1, 2 and 3) were imported into the Ingenuity Pathway Analysis as described in Material and Methods. The proteins marked in red represent the upregulated proteins and in green the downregulated proteins. The complex III proteins formed a clearly defined cluster of functionally related proteins. The solid arrows represent direct interactions and the dotted arrows indirect interactions.

To further analyze the networks connecting the deregulated proteins, all deregulated proteins (Tables 1, 2 and 3) were imported into the Ingenuity Pathway Analysis (Ingenuity System, http://www.ingenuity.com); the top scoring protein interaction network representing merged pathways of lipid metabolism, small molecule biochemistry, and cellular death is shown in Figure 2. The complex III proteins formed a clearly defined cluster of functionally related proteins. The Ingenuity Pathway Analysis (IPA) report is shown in the Information S2 (IPA Report). The biological network including the most deregulated proteins was highly significant with a score of 34. The score represents the logarithm of the probability that the network would be found by chance; score ≥2 is considered significant. This network consisted of biological categories lipid Metabolism, small molecule biochemistry, and cell death. The most relevant functions extracted from this network were related to metabolic disease (4 focus proteins), cardiovascular disease (5) and genetic disorder (10).

### High-dose radiation decreased the activity of respiratory complexes I and III

**Figure 3:**
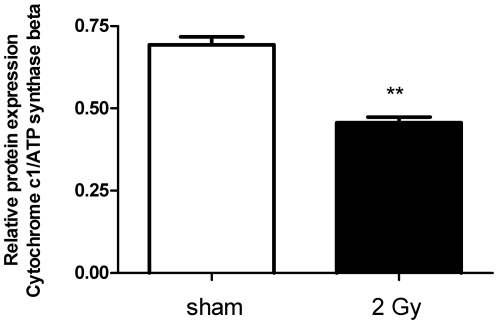
Immunoblot validation of cytochrome c1 depletion. The level of columns represent the average ratios with standard errors (SEM) of relative protein expression in sham- and 2 Gy-irradiated cardiac mitochondria. The protein bands were quantified using ImageQuant 5.2 software (GE Healthcare) by integration of all the pixel values in the band area after background correction, normalized to the ATP synthase beta subunit expression. Five biological replicates were used. **p≤0.01, t-test.

**Figure 4:**
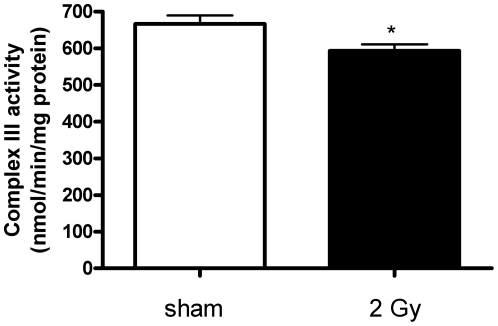
Complex III activity in 2 Gy-irradiated compared to sham-irradiated mitochondria. Complex III activity was decreased significantly (11%; p≤0.05) in irradiated samples. It was measured by the reduction of cytochrome c at 550 nm in the presence or absence of antimycin (Complex III inhibitor). Four independent biological replicates were used. *p≤0.05, t-test. The error bars represent standard error (SEM).

Cytochrome c1 is a subunit of cytochrome b-c1 complex (Complex III) in the ETC transferring electrons to cytochrome c in Complex IV. Immunoblot analysis confirmed the proteomics data showing downregulation of cytochrome c1 by 34% (p≤0.05; Figure 3; Information S1). The reduced levels of cytochrome c1 were accompanied with a significant decrease in Complex III activity by 12% in 2 Gy-irradiated hearts compared to sham-irradiated hearts (p≤0.05; Figure 4).

**Figure 5:**
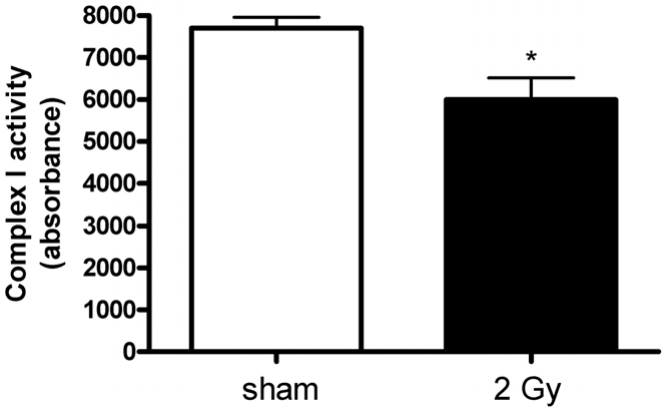
Complex I activity in 2 Gy-irradiated compared to sham-irradiated mitochondria. Complex I activity was decreased significantly (32%; p<0.05) in irradiated samples. It was measured by the dipstick assay kit as described in Experimental Section. Four independent biological replicates were used. *p≤0.05, t-test.

In accordance with the ICPL data we found the Complex I activity to be significantly downregulated by 32% (p<0.05; Figure 5).

**Figure 6:**
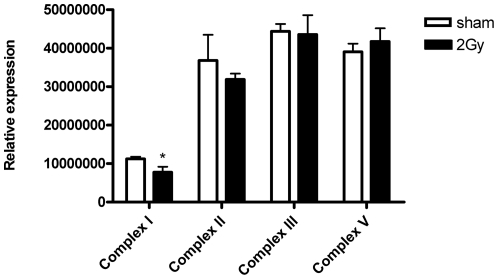
A graphical presentation of immunoblotting analysis of respiratory chain complexes. To compare the amounts of Complexes I to V in sham vs. irradiated mitochondria a premixed cocktail including one antibody against each complex was used: CI subunit NDUFB8, CII-30 kDa, CIII-Core protein 2, CIV subunit I and CV alpha subunit. CI subunit NDUFB8 was significantly decreased (29%; p≤0.05) in 2 Gy- vs. sham-irradiated cardiac mitochondria. CIV subunit I was not detected, irrespectively whether the samples were heated or not prior to the immunoblotting analysis. Four independent biological replicates were used for statistical analysis. *p≤0.05, t-test.

To further analyze the complexes of ETC, we used a premixed cocktail including antibodies against proteins important for the assembly of each complex as explained in Experimental Procedures. In this manner the relative levels of all 5 OXPHOS complexes could be measured (Figure 6; Information S1). We were able to detect the CI subunit NDUFB8, CII-30 kDa, CIII-Core protein 2, and CV alpha subunit. CIV subunit I was not detected, irrespectively whether the samples were heated or not prior to the immunoblotting analysis. The level of Complex I was significantly downregulated (29%; p≤0.05). There were no significant differences in protein levels of complexes II, III and V between 2 Gy- and mitochondria from-sham irradiated mice.

### Succinate-driven respiration was impaired in 2 Gy-irradiated mitochondria

**Figure 7:**
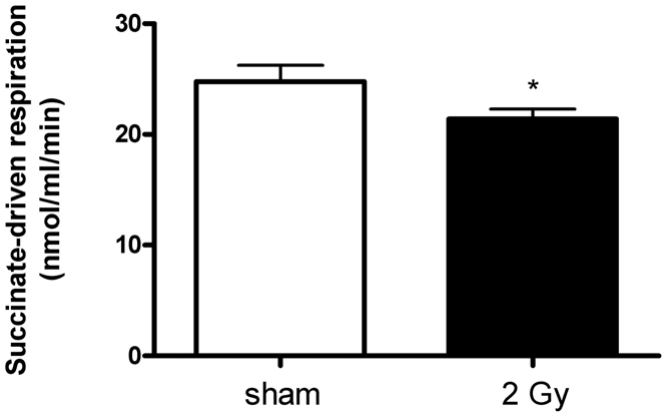
Succinate-driven respiration of the heart mitochondria from sham- and 2 Gy-irradiated hearts. Succinate-driven respiration was decreased 13% significantly (p≤0.05) in 2 Gy-irradiated mitochondria compared to sham-irradiated samples. Statistical calculations were performed using 4 independent biological replicates. *p≤0.05, t-test.

Mitochondrial respiration was measured using either succinate or palmitoylcarnitine as the substrate to analyze the intactness of mitochondria and to determine the efficiency of oxygen consumption. Succinate is a direct substrate of the respiratory chain, feeding directly into complex II, and is therefore used to determine the functionality of the ETC. Succinate–stimulated respiration (state 2 respiration) decreased significantly by 13% in mitochondria from mouse hearts irradiated at 2 Gy (Figure 7). Palmitoyl-driven respiration showed a similar trend in cardiac mitochondria isolated from mice irradiated with 2 Gy (12% decrease) but this result did not reach significance (Information S1). No significant alteration was found in the succinate-driven respiration after irradiation with 0.2 Gy (Information S1). No significant change was observed in the ADP-dependent respiration (Information S1).

### The level of ROS was enhanced in high-dose irradiated mitochondria

**Figure 8:**
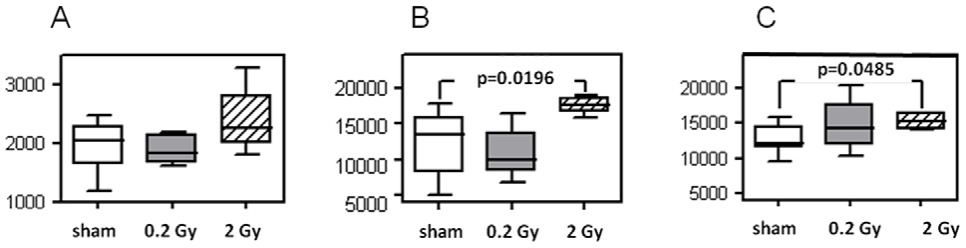
ROS/RNS production in sham-irradiated, 0.2 Gy-, and 2.0 Gy-irradiated cardiac mitochondria in the presence of succinate (8A) and stressors 50 mM mercury (8B) or 50 mM tert-butyl-COOH (8C). Mitochondria were incubated with dichlorodihydrofluorescein diacetate (DCF) and reaction was followed by measuring fluorescence (ex 485/20, em 528/20 nm) at room temperature for 60 min. In the presence of succinate alone, 2 Gy cardiac mitochondria showed a non-significant tendency of increase in ROS/RNS production. The further addition of 50 µM mercury or 100 µM *tert*-butyl-COOH induced significantly (p≤0.05, Mann Whitney test) the ROS/RNS production in 2 Gy-irradiated but not in 0.2 Gy-irradiated cardiac mitochondria compared to sham-irradiated mitochondria. Statistical calculations were performed using at least five independent biological replicates.

To analyze the ROS production, mitochondria were labeled using dichlorofluorescein (DCF). In the presence of 5 mM succinate, a substrate for succinate dehydrogenase (Complex II), a non-significant increase in ROS formation in 2 Gy-irradiated vs. sham-irradiated mitochondria was observed (Figure 8A). We tested the ROS production in the presence of additional stress factors such as mercury and *tert*-butyl-OOH that are known to impair the process of oxidative phosphorylation, cause a decline in both transmembrane potential and intracellular pH, and induce the production of ROS [63], [64]. Figure 8 (B, C) shows ROS formation after incubation of mitochondria with DCF in the presence of 50 µM mercury and 100 µM *tert*-butyl-OOH, respectively. Both mercury and *tert*-butyl-OOH induced a significant increase in ROS production in 2 Gy-irradiated vs. sham-irradiated mitochondria.

**Figure 9:**
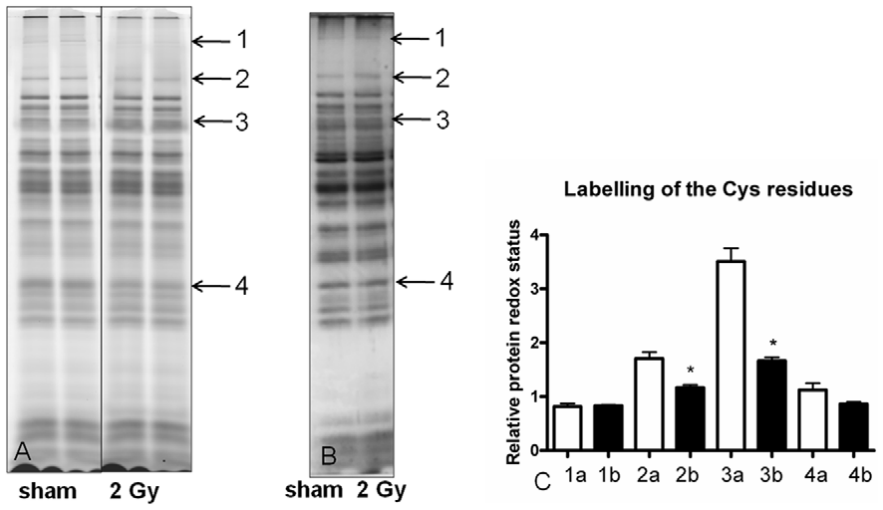
General oxidative status of the sham- vs. 2 Gy- irradiated mitochondrial proteomes. The level of oxidized proteins was analyzed by saturation DIGE labeling (A). The bands 1–4 were chosen for further analysis as they showed different intensities between sham and exposed samples after the saturation DIGE labeling. The normalization was done by comparing the DIGE band intensities to the total amount of silver stained protein bands (B). In the graphical presentation (C) the columns marked “a” represent bands from the sham-irradiated sample and the columns marked “b” represent bands from irradiated samples. Bands 2 and 3 are significantly downregulated in irradiated samples thus representing oxidized protein groups. Two biological replicates are shown. *p≤0.05, t-test, n = 3.

To identify *in vivo* targets of the increased mitochondrial ROS, the general oxidative status of the sham- vs. 2 Gy- irradiated mitochondrial proteomes was analyzed by saturation DIGE labeling (Figure 9A) that targets specifically the thiol groups of cysteine residues in the protein. We found several bands, the intensity of which was significantly decreased in the irradiated mitochondria, indicating an increased oxidation of mitochondrial proteins. The amount of the oxidized proteins was normalized to the total amount of proteins measured by post-stained silver gels (Figure 9B). Two bands were found to be significantly more oxidized after the normalization (Figure 9C).

In addition, we tested the activity of aconitase, an enzyme known to be vulnerable to oxidative stress. Aconitase activity showed decreasing tendency in irradiated samples but it did not reached significance (p≤0.15; Information S1).

### Radiation induced the activity of pyruvate dehydrogenase E1αby dephosphorylation

**Figure 10:**
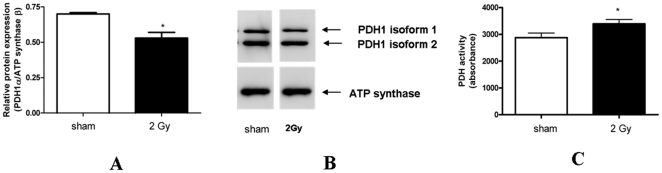
The protein amount and activity of pyruvate dehydrogenase. Immunoblot analysis confirmed the 2D-DIGE data showing that protein level of pyruvate dehydrogenase E1α was significantly decreased by 33% (*p≤0.05, t-test). Columns represent the average ratios of relative protein expression in sham- and 2 Gy-irradiated cardiac mitochondria (Figure 10A). The protein bands were quantified using ImageQuant 5.2 software (GE Healthcare) by integration of all the pixel values in the band area after background correction, normalized to the ATP synthase beta subunit expression. The protein appeared in two isoforms, one of which was significantly downregulated by radiation (upper band on the blot). Five biological replicates were used (Figure 10B). The PDH activity was measured by the dipstick assay kit as described in Experimental Procedures (Figure 10C). It was significantly enhanced in the irradiated mitochondria by 18% (*p≤0.05, t-test). Three biological replicates with 5 technical replicates each were used.

2D-DIGE approach showed a dose-independent downregulation of PDH E1α. Immunoblotting analysis confirmed the 2D-DIGE data showing that protein level was significantly decreased by 33% (p≤0.05; Figure 10A). LC/MS/MS identification of the downregulated 2D-DIGE spot identified the serine-containing peptides as phosphorylated (Information S2). Also the immunoblot showed two isoforms, only the upper band being downregulated (Figure 10B). As the dephosphorylated form of PDH is known to be active, we measured the enzyme activity; it was significantly upregulated by 18% (Figure 10C). Using ICPL technology we found no significant deregulation of this protein.

### Mitochondrial morphology was not altered by irradiation

Electron microscopic examination of mitochondria isolated from both sham- and 2 Gy irradiated hearts revealed a high degree of mitochondrial intactness and integrity of mitochondrial cristae (Information S1). The mitochondria of irradiated hearts showed a trend to a smaller size but this did not reach significance.

## Discussion

Epidemiological data indicate an association between increased risk of cardiovascular disease and enhanced oxidative stress in populations exposed to ionizing radiation (7–15). A sound functioning of the heart is strongly dependent on the energy supply provided by oxidative metabolism, and can be easily disturbed by mitochondrial dysfunction [60], [65], [66]. Suggesting that radiation-induced oxidative stress is involved in the development of cardiac damage, it may be reflected in the mitochondrial status of the exposed heart. In this study, we investigated whether cardiac mitochondria show alterations caused by heart-focused X-ray exposure four weeks after the irradiation.

We used two complementary proteomic approaches to study the proteome responses of cardiac mitochondria four weeks after exposure to ionizing radiation. ICPL approach revealed in depth global proteomic changes with up to 421 quantified proteins. On the other hand, 2D-DIGE revealed additional changes of protein isoforms and protein fragments as well as posttranslational modifications. We found that, using 2D-DIGE, the level of PDH E1α is significantly down-regulated with both radiation doses (Figure 1, Table 3). The PDH complex is a nuclear-encoded mitochondrial multienzyme complex that catalyzes the overall conversion of pyruvate to acetyl-CoA and CO_2_, and provides the primary link between glycolysis and the tricarboxylic acid (TCA) cycle. The PDH complex is composed of multiple copies of three enzymatic components: pyruvate dehydrogenase (E1), dihydrolipoamide acetyltransferase (E2) and lipoamide dehydrogenase (E3). The E1α component has three phosphorylation sites that in large part regulate the PDH activity, phosphorylation leading to inactivation [67]. Confirming the 2D-DIGE data, immunoblotting against PDH E1α revealed two bands, the high molecular weight isoform showing downregulation (Figure 10 A, B). The PDH activity was significantly up-regulated by 18% (Figure 10 C). We propose that the increased activity is due to the downregulation of the phosphorylated form of E1α. Our LC/MS/MS identification of the downregulated 2D-DIGE spot identified the serine-containing peptides as phosphorylated (Information S2); these are the phosphopeptides known to be responsible for the PDH activity [68]. The increased PDH activity suggests an enhancement of substrate flow to the TCA cycle and reduction in the lactate formation.

We show that mitochondrial respiration was significantly decreased in hearts exposed to 2 Gy radiation compared to sham-irradiated hearts when succinate was used as substrate (Figure 7). Succinate is a direct substrate for ETC, feeding electrons into the Complex II. The observed decrease in respiration capacity indicates an impaired function in the respiratory chain that is confirmed by the proteomics analysis (Tables 1 and 3). No significant change was observed in the ADP-driven respiration *in vitro* (Information S1).

A decrease in the mitochondrial respiration rate has been reported to increase ROS production [61]. In mammalian cells, including cardiac myocytes, the mitochondrial ETC is the major source of ROS production [69], [70]. Low endogenous levels of ROS play an important role in modulating cell signaling pathways but increased levels of ROS are known to induce cell death [71], [72].

We show that, in the presence of stressors known to have inhibitory effects on the antioxidant system [63], [64], a significant increase in ROS production was observed *in vitro* in mitochondria from 2 Gy-exposed mice (Figure 8). This suggests that irradiated mitochondria are less able to deal with additional stress factors. Indeed, synergistic adverse effects of metals and radiation have been observed previously. The presence of non-toxic concentrations of mercury has been shown to potentiate the effect of low-dose ionizing radiation by disturbing brain development in neonatal mice whereas neither agent alone had an effect [73].

Mitochondrial ROS is mainly produced by Complex I and Complex III in the presence of a reverse flow of electrons from succinate dehydrogenase (Complex II). We show here that the activity of both complexes is significantly decreased by radiation by 32% (Complex I) (Figure 5) and 11% (Complex III) (Figure 4). Bearing in mind that in mitochondria from failing hearts the Complex III activity was decreased by 26% [61], the radiation-induced activity reduction seen here will probably play a major biological role.

The 2D-DIGE approach and immunoblotting showed radiation-induced down-regulation of cytochrome c1 (Table 3, Figure 3). It is the heme-containing component of the cytochrome b-c1 complex, the last component in ETC in Complex III, accepting electrons from Rieske protein and transferring them to cytochrome c of Complex IV. As the assembly of Complex III, measured as the level of Core protein 2, was not altered (Figure 6), we conclude that the decreased activity of this complex is mainly due to the reduced level of cytochrome c1. Importantly, we are able to show that the radiation-induced increase in endogenous ROS targets the mitochondrial proteome, seen as an enhanced level of oxidized proteins (Figure 9).

We identified four structural proteins, actin, myosin-6, desmin and vimentin that, in contrast to many metabolic enzymes, were up-regulated after radiation exposure. Heart mitochondria contain the highest percentage of non-mitochondrial proteins, mainly structural proteins such as myosin and actin [74]. The association of mitochondria with the cytoskeleton has been known for many years and numerous studies suggest that the cytoskeleton is involved in movement and localization of mitochondria [75], [76]. Vimentin and desmin are intermediate filaments that function in structural support, signal transduction and organelle positioning in the cell. Several recent findings suggest that these filaments maintain mitochondrial morphology and organization and support mitochondrial function [77]–[79]. Inactivation of the desmin gene in mice heart tissue resulted in mitochondria showing abnormal shape, distribution and function [80]. It has been suggested that desmin functions to link mitochondria to myofibrils and provides a mechanism by which contractile activity influences the metabolic function of mitochondria [81]. Interestingly, vimentin has been shown to have a protective role against oxidative stress-induced damage [82]. The up-regulation of structural proteins seen here may be explained by a stronger association between cytoskeleton and irradiated mitochondria. Further studies are needed to clarify this.

The radiation-induced effects on cardiac mitochondria resemble but are not similar to hibernating mitochondria that are typical for myocardium with regional contractile dysfunction. As in our study, mitochondrial respiration is depressed in chronic hibernating myocardium [83]. Several mitochondrial protein classes (PDH complex, Complex I, structural proteins such as actin, myosin, desmin, and vimentin) are deregulated in a similar way in a hibernating swine myocardium as in our mouse model [84]. However, hibernating mitochondria show decreased PDH activity and lowered ROS production that distinguishes the radiation-induced changes from the hibernating phenotype [84].

Based on our data we suggest that initial mitochondrial dysfunction leads to increasing disbalance of the heart redox status. Our recent data using total body irradiation (3 Gy gamma-ray) shows that mitochondrial damage occurred within hours after irradiation and that the mitochondria were the organelles most sensitive to radiation and thus a direct target of radiation damage [42]. In addition, irradiation caused an immediate increase in the levels of protein oxidation and lipid peroxidation.

We suggest that an altered redox status will result in impaired heart function and/or increased vulnerability towards additional stress conditions in the long term. In line with this hypothesis Fedorova, et al. showed that total body irradiation (5 Gy X-ray) induced preferential oxidation of myofibrillar proteins with intra- and intermolecular disulphide bridges between actin and various isoforms of myosin light chain [85]. As mitochondria are closely associated with myofibrils it is reasonable to suggest that increased mitochondrial ROS production may lead to impaired contractility through disruption of actin-myosin interactions [86].

In conclusion, we show here that a local dose of 2 Gy results in both functional and proteomics alterations in cardiac mitochondria whereas using the 0.2 Gy dose only a few alterations can be observed in the mitochondrial proteome and no effect is seen in the mitochondrial function. Although there seems to be a dose-dependent enhancement in the total number of deregulated proteins, it is too early to make a statement about the possible increase in the risk for CVD with doses lower than 0.5 Gy. Consequently, the discrepancy seen in the epidemiological data remains unsolved. To elucidate the progression of the radiation-induced damage with low doses we intend to investigate later time points such as 40 weeks after irradiation.

With higher doses (2 Gy) ionizing radiation causes non-transient mitochondrial alterations in three major biological categories: the pyruvate metabolism, the oxidative phophorylation and the mitochondria-associated cytoskeleton. The changes in the pyruvate metabolism and structural proteins are seen with both low and high radiation doses. Our data confirm that the radiation-induced impairment of the respiratory chain is tightly coupled to increased ROS levels in the heart and is reflected as increased protein oxidation. This may contribute to cardiac remodelling seen here as alteration of the structural proteins and simultaneously serve as a first stage in the etiology of radiation-induced heart disease.

## Supporting Information

Information S1 **Verification of mitochondrial purity, quantification of protein standards by ICPL, gel pictures, functional respiratory measurements, aconitase activity and mitochondrial electron microscopic images.**
(PDF)

Information S2 **LC/MS-MS Experimental Settings, mass spectrometry identification of 2D spots, ICPL quantification data (Proteome Discoverer 1.2) and Ingenuity Pathway Analysis.**
(XLS)
