# Supplementary material for: Radiation–Induced Signaling Results in Mitochondrial Impairment in Mouse Heart at 4 Weeks after Exposure to X-Rays
Source: PLoS One. 2011 Dec 8;6(12):e27811. doi: 10.1371/journal.pone.0027811 (PMC3234240; doi:10.1371/journal.pone.0027811)
Supplement: Information S1 — Verification of mitochondrial purity, quantification of protein standards by ICPL, gel pictures, functional respiratory measurements, aconitase activity and mitochondrial electron microscopic images. (PDF) [file pone.0027811.s001.pdf]

Supplemental File 1

Radiation–induced Signaling Results in Mitochondrial Impairment in Mouse Heart at 4 Weeks after Exposure to X-rays

Zarko Barjaktarovic[1]*, Dominik Schmaltz[1], Alena Shyla[1], Omid Azimzadeh[1], Sabine Schulz[2], Julia Haagen[3], Wolfgang Dörr[3], Hakan Sarioglu[4], Alexander Schäfer[4], Michael J. Atkinson[1, 5], Hans Zischka[2], and Soile Tapio[1]

[1]Institute of Radiation Biology, Helmholtz Zentrum München, German Research Center for Enviromental Health, Ingolstaedter Landstrasse 1, 85764 Neuherberg, Germany; *[2]Institute of Toxicology, Helmholtz Zentrum München, German Research Center for Enviromental Health*; *[3]Department of Radiotherapy and Radiooncology, Medical Faculty Carl Gustav Carus, University of Technology, Dresden, Germany*; *[4]Helmholtz Zentrum München, German Research Center for Environmental Health, Department of Protein Science, Proteomics Core Facility, 85764 Neuherberg, Germany;[5]Department of Radiation Oncology, Klinikum Rechts der Isar, Technische Universität München, Munich, Germany*

*Corresponding author:

Zarko Barjaktarovic

Tel. +49(0)89 3187 3855

Fax +49(0)89 3187 3378

zarko.barjaktarovic@helmholtz-muenchen.de

Key words: Cardiac mitochondria, ionizing radiation, mitochondrial complexes, reactive oxygen species, proteomics, Complex I, Complex III

Table S1. Quantification of the ICPL standards

Accession UniProt	Protein name	Σ# Peptides	Heavy/ Light measured	Heavy/ Light theoretical	Heavy/ Light Counts	Heavy/Light Variability [%]	Relative Error (%)
CAH2_BOVIN	Carbonic anhydrase II	5	2,08	2,0	8;10;7	0,6	4,0
ALBU_BOVIN	Serum albumin	8	0,93	1,0	17;18;7	20,2	7,0
OVAL_CHICK	Ovalbumin	10	0,244	0,25	19;19;10	5,3	2,4

A

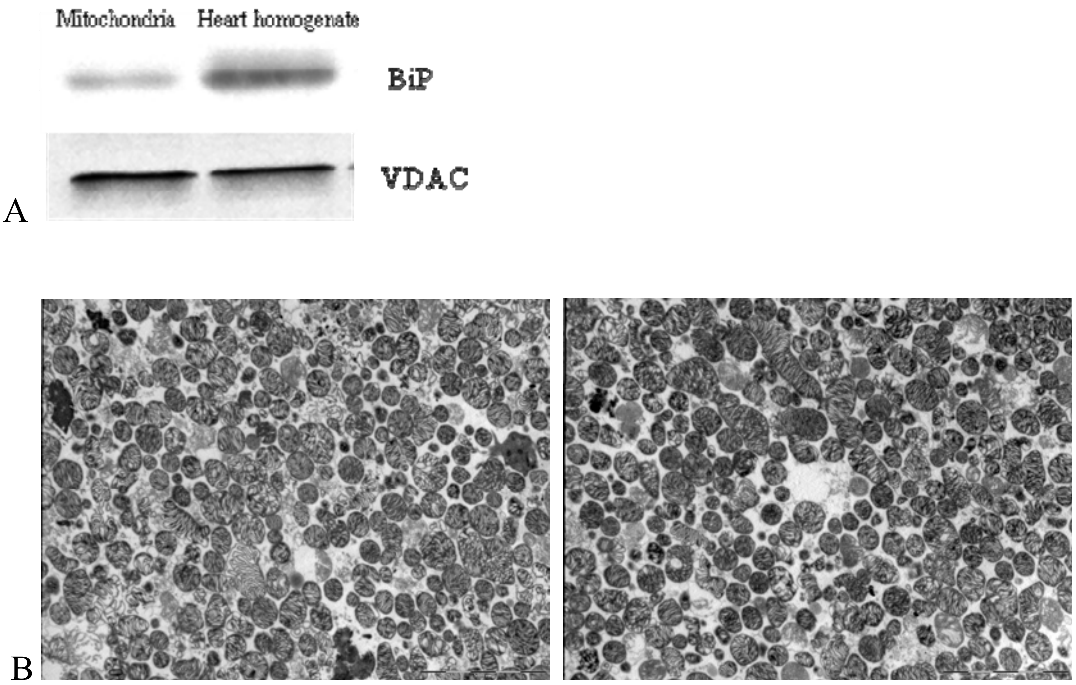

B

Figure S1. Verification of mitochondrial purity. A) Western Blot analysis using markers for mitochondria (VDAC) and ER (BiP) is shown. B) Electron microscopy images of purified mitochondrial fractions; magnification 5000X.

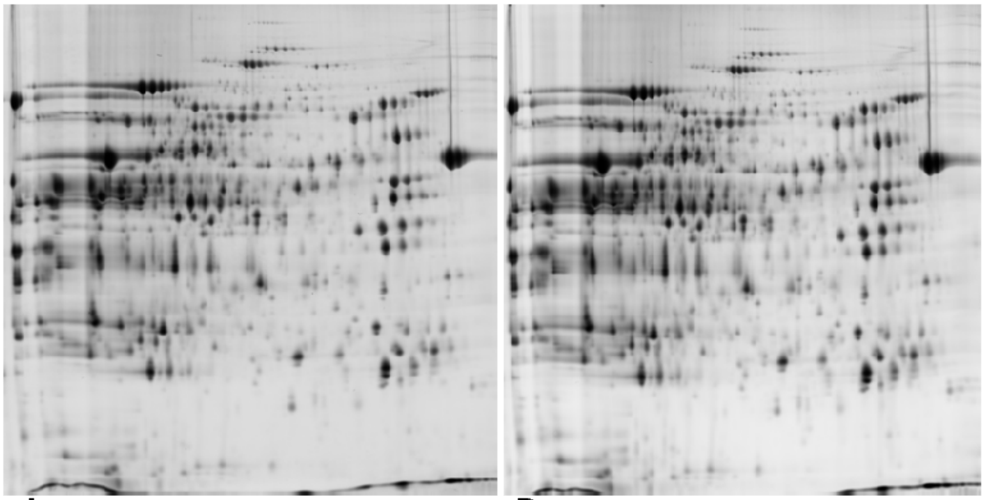

A B

Figure S2. Two dimensional separation of mitochondrial proteins 4 weeks after irradiation. Sham (A) and 2 Gy (B) irradiated samples are shown.

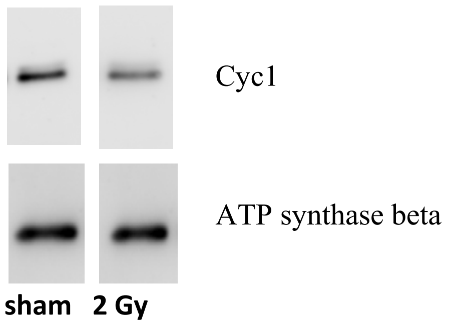

Figure S3. Western blot of cytochrome c1 in sham- and 2 Gy-irradiated mitochondria. ATP synthase was used as loading control. The level of columns represent the average ratios with standard errors (SEM) of relative protein expression in sham- and 2 Gy-irradiated cardiac mitochondria. The protein bands were quantified using ImageQuant 5.2 software (GE Healthcare) by integration of all the pixel values in the band area after background correction, normalized to the ATP synthase beta subunit expression. Five biological replicates were used. **p≤ 0.01, t-test

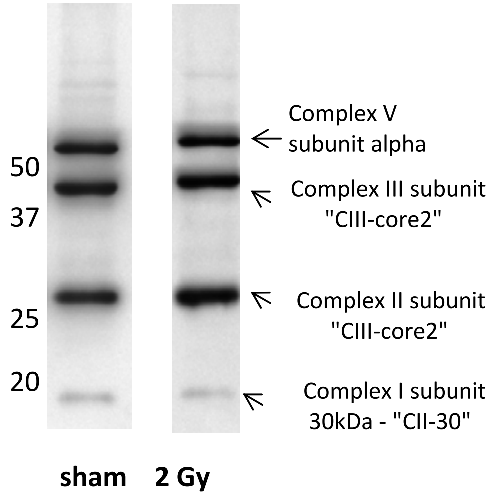

Figure S4. Western blot analysis of respiratory chain complexes. To compare the amounts of Complexes I to V in sham vs. irradiated mitochondria a premixed cocktail including one antibody against each complex was used: CI subunit NDUFB8, CII-30kDa, CIII-Core protein 2, CIV subunit I and CV alpha subunit. CI subunit NDUFB8 was significantly decreased (29%; $p \leq 0.05$) in 2 Gy- vs. sham-irradiated cardiac mitochondria. CIV subunit I was not detected, irrespectively whether the samples were heated or not prior to the immunoblotting analysis. Four independent biological replicates were used for statistical analysis.

*$p \leq 0.05$, t-test

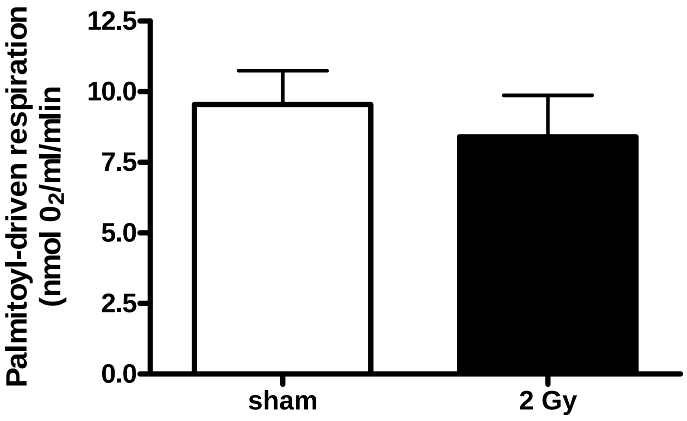

Figure S5. Palmitoyl carnitine-driven respiration of the heart mitochondria from sham- and 2 Gy-irradiated hearts. Palmitoyl carnitine -driven respiration was decreased 12% in 2 Gy-irradiated mitochondria compared to sham-irradiated samples but this was not statistically significant. Statistical calculations were performed using 3 independent biological replicates.

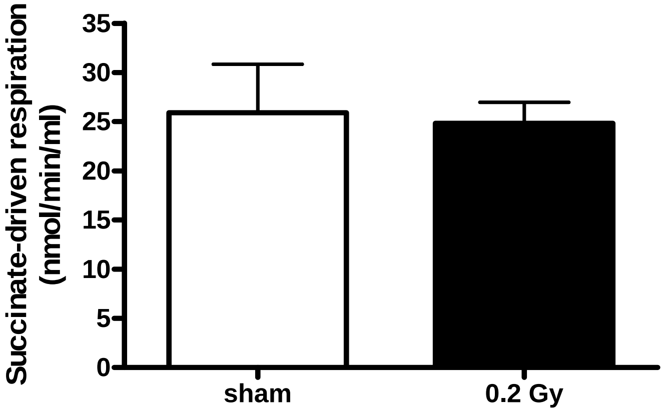

Figure S6. Succinate-driven respiration of the heart mitochondria from sham- and 0.2 Gy-irradiated hearts. There was no significant change between the irradiated and sham-irradiated mitochondria. Statistical calculations were performed using 4 independent biological replicates.

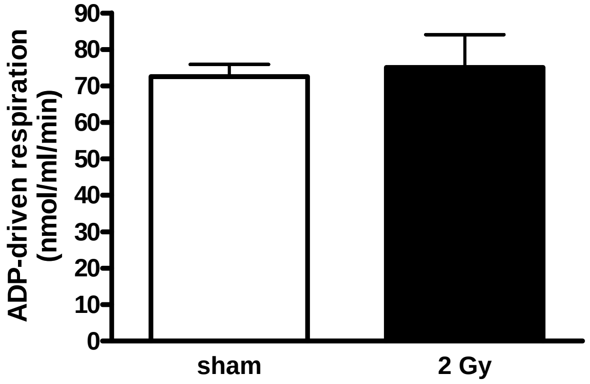

Figure S7. ADP-driven respiration of the cardiac mitochondria from sham- and 2 Gy-irradiated hearts. No significant change was observed in the state 3 respiration of cardiac mitochondria irradiated with 2 Gy when compared to sham-irradiated samples. Statistical calculations were performed using 4 independent biological replicates.

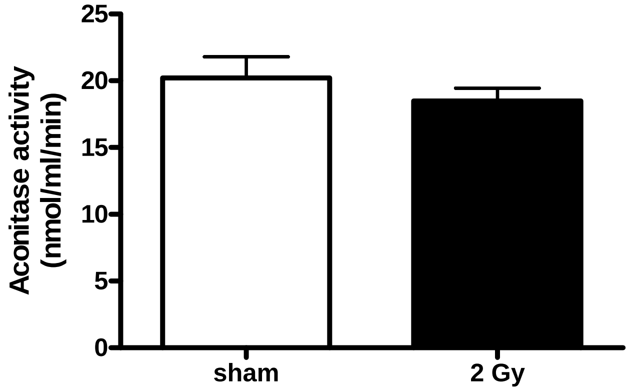

Figure S8. Aconitase activity in 2 Gy- and sham-irradiated cardiac mitochondria. The aconitase activity was measured as the increase in absorbance at 340 nm. Aconitase activity was decreased 8% but the result did not reach significance ($p \leq 0.15$). Statistical calculations were performed using 4 independent biological and 2 technical replicates.

.

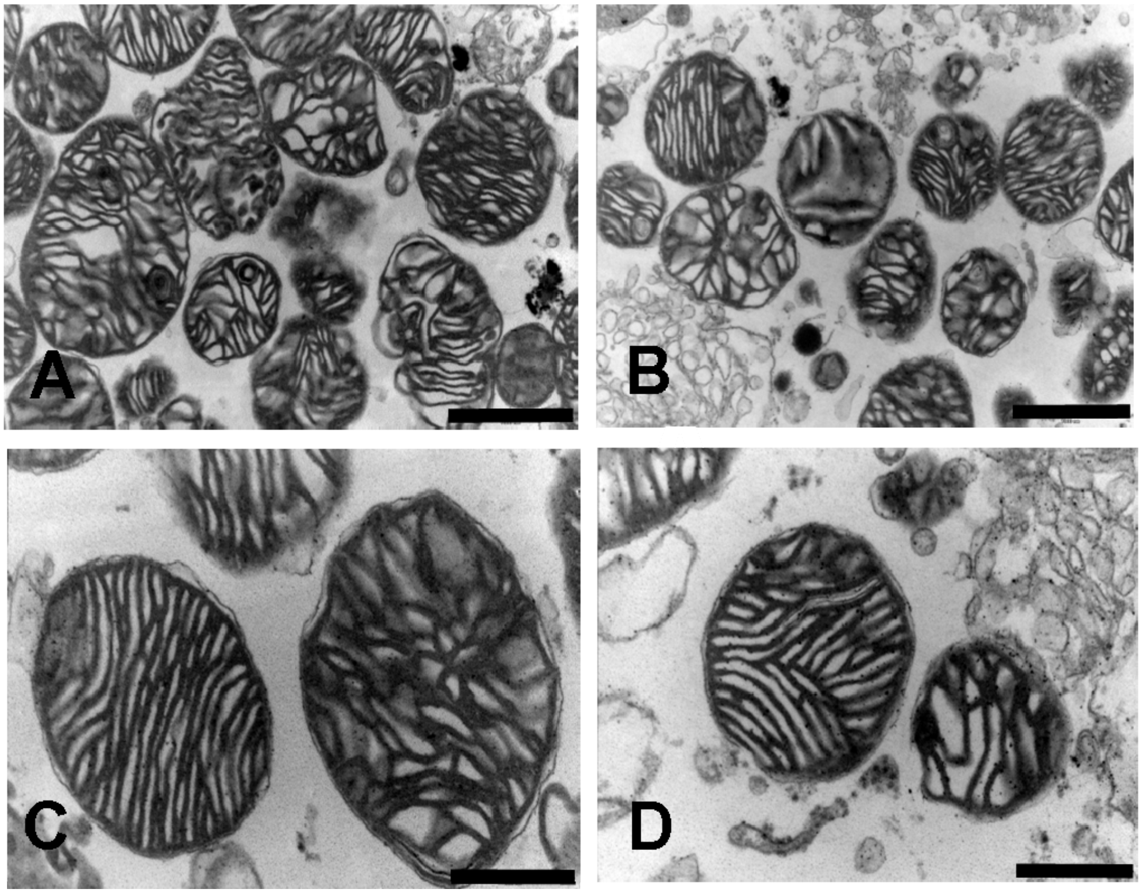

Figure S9. Electron microscopic images of mitochondria isolated from sham-irradiated (A) and 2 Gy-irradiated (B) hearts. EM images revealed a high degree of mitochondrial intactness and integrity of mitochondrial cristae that was not affected four weeks after exposure. The mitochondria of irradiated hearts showed a trend to a smaller size but this did not reach significance. A. Mitochondria isolated from sham-irradiated mice; magnification 25.200. The size bar corresponds to 1.000 nm. B. Mitochondria isolated from 2 Gy-irradiated mice; magnification 25.200. The size bar corresponds to 1.000 nm. C. Mitochondria isolated from sham-irradiated mice; magnification 50.000. The size bar corresponds to 500 nm. D. Mitochondria isolated from 2 Gy-irradiated mice; magnification 50.000. The size bar corresponds to 500 nm. Courtesy of Prof. Dr. Walch, Helmholtz Zentrum München.
